# Burden of CIN2+ diagnoses and conizations in women aged 18–45 years—a retrospective secondary data analysis of German statutory health insurance claims data

**DOI:** 10.1007/s00404-022-06548-7

**Published:** 2022-04-14

**Authors:** Miriam Reuschenbach, Anna-Janina Stephan, Kunal Saxena, Vimalanand S. Prabhu, Christian Jacob, Kim Maren Schneider, Wolfgang Greiner, Regine Wölle, Monika Hampl

**Affiliations:** 1 Global Medical and Scientific Affairs, MSD Sharp & Dohme GmbH, Levelingstr. 4a, 81673 Munich, Germany; 2 Department of Market Access, MSD Sharp & Dohme GmbH, Munich, Germany; 3 Center for Observational and Real-World Evidence (CORE), Merck & Co., Inc., Kenilworth, NJ USA; 4 EU Real World Evidence, Xcenda GmbH, Hanover, Germany; 5 Department of Health Economics and Health Care Management, Bielefeld School of Public Health, Bielefeld University, Bielefeld, Germany; 6 Department of Gynecology, University of Duesseldorf, Duesseldorf, Germany

**Keywords:** Cervical conization, Cervical intraepithelial neoplasia (CIN), Claims data analysis, Cervical cancer screening, HPV, Germany

## Abstract

**Purpose:**

High grade cervical intraepithelial neoplasia (CIN2+) may progress to cervical cancer. They may be detected by screening and are usually treated by conization. This study aimed at assessing annual proportions of screening, prevalent and incident CIN2+ diagnoses, as well as proportions of (re-)conizations during 24 months follow-up after conization in Germany.

**Methods:**

A descriptive retrospective claims data analysis of the years 2013–2018 was conducted using the InGef Research Database. Women aged 18–45 years with CIN2+ diagnoses were identified by ICD-10-GM codes (N87.1, N87.2, D06.-, and C53.-). Cervical conizations were identified by OPS codes (5–671.0* or 5–671.1*). Screening participation was identified by EBM codes (01730, 01733, 32819 or 32820). Annual proportions were calculated as women with the respective documented codes divided by all women in the respective age group per calendar year.

**Results:**

Overall annual proportions of screened women spanned from 60.01 to 61.33% between 2013 and 2018. The overall annual prevalence of CIN2+ diagnoses (regardless of screening participation) ranged from 0.72 to 0.84% between 2013 and 2018, with highest proportions observed in women aged 27–45 years. Also, CIN2+ incidence was highest in women 27–45 years. Annual proportion of women undergoing conization was 0.24% in 2013 and 0.21% in 2018. During a 24-month follow-up period after conization, 2.91% of women underwent a re-conization 3 months or later after the initial conization.

**Conclusion:**

This analysis demonstrates a considerable burden of CIN2+, conizations and re-conizations in Germany, especially in women aged 27–45 years. This highlights the need for intensified prevention efforts such as expanding human papillomavirus (HPV) vaccination.

**Supplementary Information:**

The online version contains supplementary material available at 10.1007/s00404-022-06548-7.

## Background

Cervical intraepithelial neoplasia (CIN) is a potential consequence of human papillomavirus (HPV) infection. Although oncogenic HPV infection of the uterine cervix is very common and may be acquired throughout life [1], the peak prevalence in most European countries in women is below the age of 25 years [2]. In Germany, cervical HPV infection prevalence in 25–26-year-old women was around 23% in 2009–2010 [3]. While around 90% of cervical HPV infections clear naturally over a few months to years, in about 10% of cases, infections persist [4]. In about 5% of women with persistent cervical HPV infection, high-grade CIN (CIN2 and CIN3) may develop over a period of 1–3 years [5–7]. If left untreated, high grade CIN, which are considered precancerous lesions, may progress to cervical cancer [8].

In Europe, about 45% of CIN2+ and 70% of cervical cancers are attributable to HPV genotypes 16 and 18 and more than 80% of CIN2+ and 90% of cervical cancers to genotypes 16, 18, 31, 33, 45, 52, 58 [9]. A recent German study evaluated the distribution of high-risk HPV types in women with CIN and cervical cancer [10]. HPV genotypes 16 or 18 were detectable in 40% of CIN3 lesions and 79% of cervical cancers. All three currently licensed HPV vaccines target HPV genotypes 16 and 18. The quadrivalent and nonavalent vaccines also target HPV genotypes 6 and 11, which cause approximately 90% of anogenital warts. The nonavalent vaccine additionally protects against genotypes 31, 33, 45, 52, 58. Thus, most CIN lesions and cervical cancer cases, as well as certain other HPV-associated diseases, are preventable. HPV vaccination is an important pillar, along with cervical cancer screening and treatment of pre-cancers, of the WHO strategy to eliminate cervical cancer as a public health threat [11, 12].

In Germany, HPV vaccination, screening for precancerous lesions, and treatment of CIN is funded by the statutory health insurance (SHI). HPV vaccination was introduced in 2006 and recommended with mandatory funding in 2007 for 12–17-year-old girls until 2014 [13]. Since 2014, the recommendation was updated to 9–14-year-old girls with catch-up vaccination until the age of 17 [14] (since 2018 including boys of the same age [15]). From 1971 to 2019, an annual opportunistic Pap test screening was offered free of charge by the SHI for women aged 20 years and older [16]. HPV tests were not part of the screening and were mainly used if Pap test results were equivocal or if they were paid out of pocket. Since January 1st 2020, an organized screening program supported by individual invitation letters was introduced including annual screening for women aged 20–34 years using Pap test and co-testing (Pap test + HPV test) in 3-year intervals for women aged 35 years and older [17, 18].

Nevertheless, cervical cancer and its precursors remain present in Germany. Estimates from 2011 to 2013 [19] and 2016 [20] suggest that about 50,000 women underwent histological diagnostic examination following positive or equivocal cytology results per year, which in most cases led to a subsequent CIN diagnosis. In women born 1990, 3-year prevalence of CIN3 in Germany from 2013 to 2015 was 0.3% [21]. Furthermore, 4640 new cervical cancer cases were reported in 2012, and about 4300 women were projected to be diagnosed with cervical cancer in 2016 [22].

German guidelines for the prevention of cervical cancer recommend watching and waiting for 6–12 months and even longer in patients with CIN1/2 and to re-evaluate the patient subsequently. If the patient has received a positive result for CIN3, excision or ablation of the abnormal tissue, usually by conization, is recommended [23].

Conization of the cervix uteri (cervical conization) is a standard surgical procedure, which can be done with either scalpel, laser, or with an electrosurgical instrument typically referred to as LEEP (loop electrosurgical excision procedure) [24]. The German guideline recommends LEEP or LEETZ (loop excision of the transformation zone) or laser conization as preferred treatment of cervical precancer [23, 25]. Estimates for frequency of annual conizations in Germany vary considerably between 50,000 and 140,000 (based on 2006–2009 extrapolated estimates) [23, 26, 27].

Women diagnosed with CIN3 and treated by conization remain at increased risk of developing subsequent CIN and cervical cancer as well as other HPV-associated neoplasia [28, 29]. CIN diagnoses and treatment have been reported to present a considerable burden, and might potentially be prevented through HPV vaccination [30]. Currently, there is only sparse, sometimes inconsistent, and partially outdated data on frequency of CIN diagnoses and conization procedures in Germany.

The aim of this study was to explore proportions of women screened for precancerous lesions, the burden of CIN2+ diagnoses and conizations, as well as subsequent CIN records and re-conizations in women aged 18–45 years in a real-world setting based on German claims data for the years 2013–2018.

## Methods

### Study design

We conducted a retrospective database analysis using claims data from January 1st, 2013 to December 31st, 2018 from the “Institute for Applied Health Research Berlin GmbH” (InGef) Research Database. Among all women aged 18–45 years in this database, annual proportions of women screened for precancerous lesions, annual proportions of women with incident and prevalent CIN2+ diagnoses, and annual proportions of women undergoing cervical conizations were estimated using cross-sectional descriptive analyses. In addition, we conducted a 2-year follow-up longitudinal analysis on a sub-sample of women who had undergone conization between July 1st 2013 and December 31st 2016 to explore proportions of women with subsequent CIN records six weeks or later and subsequent (re-)conizations 3 months or later after the initial conization.

### Data source

The InGef Research Database comprises anonymized claims data of about 4 million individuals from about 60 SHI companies, thereby covering about 55% of all SHI companies in Germany [31], 5.5% of the German SHI population and 4.8% of the total German population as of 2018. The database represents the German population in terms of age and gender according to the Federal Office of Statistics [32] and has proven to have good external validity to the German population in terms of morbidity, mortality, and drug use [33]. The detailed database description can be found in Supplement, Section 1.

Claims data from the participating SHIs are joined in a specialized trust center, anonymized, and subsequently transferred to InGef. As the raw dataset is not allowed to leave the secured storage facilities, all analyses were conducted by an InGef analyst in accordance with a pre-specified study protocol.

The analysis of German SHI claims data is permitted by social law and no review by an independent ethics committee was required to conduct this study.

### Study population

**Fig. 1:**
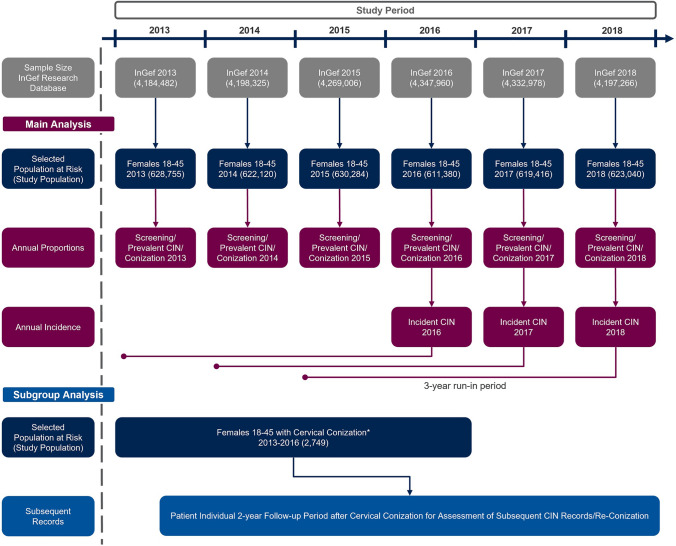
Patient selection flow chart. Abbreviations: *CIN*, cervical intraepithelial neoplasia; *InGef*, Institute for Applied Health Research Berlin. * Subsample of the same study population selected for a matched analysis with additional inclusion criteria for another part of this project which has been published elsewhere. A detailed description of the selection process of this subsample for the matching analysis can be found in the Supplement, Section 2

All women in the InGef Research Database between January 1st 2013 and December 31st 2018, who were aged 18–45 years in at least one of the respective calendar years were included in the study population (see Fig. 1 for selection process of study population).

For cross-sectional analyses of annual proportions of women screened for precancerous lesions, annual proportions of women with prevalent CIN2+ diagnoses, and cervical (re-)conizations, all women aged 18–45 years in the respective calendar year were included. All women were required to be continuously observable in the respective calendar year of analysis except for women who deceased.

For the assessment of annual proportions of women with incident CIN2+ diagnoses, the study population was restricted to women aged 18–45 years in the calendar years 2016–2018 to allow for the women’s continuous observability in the database for three calendar years prior to the year of incidence assessment. The analysis was therefore restricted to women who were continuously observable for at least four calendar years (3 years run-in period before incidence observation plus the year of interest for the respective incidence calculation).

For the assessment of subsequent CIN records as well as subsequent (re-)conizations, we used an existing subsample of the same study population selected for a matched analysis with additional inclusion criteria for another part of this study which has been published elsewhere. A detailed description of the selection process of this subsample can be found in the Supplement, Section 2. In brief, this subsample included women who underwent a conization (no re-conization, hereafter termed “index” conization) between July 1st, 2013 and December 31st, 2016 and were continuously observable 6 month before and 24 months after that conization. They were furthermore required to have at least one recorded CIN 1/2/3 diagnosis and no conization claim or cervical cancer diagnosis in the 6 months prior to the index conization.

### Variables

#### Age

For each cross-sectional analysis year, only women aged 18–45 years old in the respective year were included in the analysis. Age was determined as: analysis (calendar) year – birth year = age of the individual. Age of women assessed for analysis of subsequent CIN records and recurrent conizations was determined in the quarter of the CIN diagnosis recorded in the 6 months prior to the index conization.

#### Screening for precancerous lesions

Participation in screening for precancerous lesions was identified through claims for an HPV or Pap test, which were identified in the database through Official German Remuneration Scheme for Outpatient Care (EBM, Einheitlicher Bewertungsmaßstab) codes (see Supplement, Section 3, Supplementary Table 2). Women with at least one recorded claim of the listed EBM codes in the respective calendar year were defined as screened for precancerous lesions. The annual proportion of screened women were calculated by dividing the number of screened women in that calendar year by the total number of women in the respective calendar year in the database.

#### CIN2+ diagnoses

For the identification of CIN diagnoses we used International Statistical Classification of Diseases, 10th Revision, German Modification (ICD-10-GM) codes, which is the official classification for the encoding of diagnoses in inpatient and outpatient medical care in Germany since 2000 [34]. Clinicians in the outpatient setting are required to add one of the following specifications to the ICD-10-GM codes: “suspected diagnosis”, “diagnosis ruled out”, “condition post diagnosis”, or “verified diagnosis”. For instance, “suspected” may be coded, if the physician is not certain about the presence of the coded disease and a confirming laboratory analysis is still pending. To ensure the accuracy of diagnoses, only women with at least one outpatient “verified” diagnosis or inpatient primary or secondary diagnosis (see Supplement, Section 3, Supplementary Table 2) were considered.

The annual proportion of women with prevalent CIN2+ diagnoses (CIN2, CIN3 or cervical cancer; ICD-10 GM N87.1, N87.2, D06.-, and C53) was calculated by dividing the number of women with prevalent CIN2+ diagnoses in that calendar year by the total number of women in the study population for the respective calendar year.

Amongst those women with prevalent CIN2+ diagnoses in the calendar years 2016–2018, those without a recorded diagnosis for equal or more severe CIN grades in the three calendar years before the analysis year were considered incident CIN2+ patients. For example, a woman with CIN3 was incident with CIN3 if she had no CIN3 + diagnosis in the 3 years before the analysis year. CIN1/2 diagnoses were allowed for that case. The annual proportion of women with incident CIN2+ diagnoses in the calendar years 2016, 2017, and 2018 was calculated by dividing the number of women with an incident CIN2+ diagnosis in the respective calendar year by the total number of women in the study population who were continuously observable 3 years prior to and throughout the respective analysis year.

The annual incidence and prevalence were presented in total (CIN2+ diagnoses) and stratified by grade (CIN2, CIN3 or cervical cancer).

#### Cervical conization

For the cross-sectional analyses of annual proportion of women undergoing conization, all women with at least one recorded claim for cervical conization in the respective calendar year were identified through German classification of operation and procedures (OPS, Operations- und Prozedurenschlüssel) codes for conization and re-conization procedures (OPS 5–671.0* or 5–671.1*; see Supplement, Section 3, Supplementary Table 2 for details). The annual proportion of women undergoing cervical conization was calculated by dividing the number of women with a respective record by the total number of women in the respective calendar year.

#### Subsequent records of CIN and conization

For the longitudinal subgroup analysis of subsequent CIN records and subsequent (re-)conizations following an index conization, the index conization was defined by OPS codes for conization only (5–671.0*). Re-conization OPS codes (5–671.1*) were not used to define an index event. Subsequent CIN records and (re-)conizations were analyzed in the individual 24-month follow-up period after index conization.

CIN (1/2/3 +) records were defined as “subsequent” if they were recorded at least six weeks, i.e., from day 43 and onwards, after an index conization. As outpatient diagnoses are only available on a quarterly basis, outpatient diagnoses were considered “subsequent” if a recorded EBM code indicated that a physician visit had taken place from day 43 onwards after the index conization at the same physician who recorded the respective “verified” CIN (1/2/3 +) diagnosis in the same quarter. Inpatient primary and secondary diagnoses were considered if the date of admission was recorded from day 43 onwards after the index conization.

Subsequent cervical conizations were identified by OPS codes for conization and re-conization (see Supplement, Section 3, Supplementary Table 2). Re-conizations were considered only after a wash-out period of 90 days (i.e., 3 months) after the index conization (the date of the index conization marks day 0). The 90 days wash-out period was deemed appropriate to reflect true histologically indicated re-conizations because the first post-conization cytology is usually done after approximately 3 months.

### Statistical methods

Descriptive statistics in this study comprised frequencies and percentages for categorical variables. For continuous variables mean, standard deviation, minimum, 25th percentile, median, 75th percentile, and maximum were calculated. For the annual proportions reported in the cross-sectional analyses 95% confidence intervals were additionally reported. Results were presented both in tabular form as well as graphically using bar plots stratified by calendar year.

### Stratification and sensitivity analyses

All analyses were additionally reported stratified by age groups. For age-stratified analyses, women were categorized into the following age-groups: 18–19 years, 20–26 years, 27–30 years, 31–35 years, 36–40 years, and 41–45 years.

As CIN are usually asymptomatic and therefore predominantly identified through screening, an additional sensitivity analysis was performed. For this sensitivity analysis, assessment of annual proportions of women with prevalent and incident CIN2+ diagnoses, as well as proportions of women undergoing conization were repeated restricting the study population to screened women only instead of all women in the database. Details for the sensitivity analyses can be found in the Supplement, Section 6.

## Results

### Study population

The overall study population of women aged 18–45 years in the database was 628,755 (2013), 622,120 (2014), 630,284 (2015), 611,380 (2016), 619,416 (2017), and 623,040 (2018), respectively. A description of the participant selection flow for the different cross-sectional and longitudinal analyses can be found in Fig. 1.

Table 1 shows the age distribution of the overall study population of women aged 18–45 years in the InGef Research Database for the years 2013–2018.

**Table 1:** Age distribution of the overall study population

Age (in years)	Calendar year					
	2013	2014	2015	2016	2017	2018
Women in the database (total), *n* (%)						
18–45	628,755 (100.00)	622,120 (100.00)	630,284 (100.00)	611,380 (100.00)	619,416 (100.00)	623,040 (100.00)
Women in the database per age group, *n* (%)						
18–19	34,962 (5.56)	35,486 (5.70)	37,657 (5.97)	36,114 (5.91)	35,632 (5.75)	36,097 (5.79)
20–26	142,624 (22.68)	141,029 (22.67)	144,046 (22.85)	136,838 (22.38)	137,455 (22.19)	136,901 (21.97)
27–30	86,916 (13.82)	88,179 (14.17)	92,205 (14.63)	91,457 (14.96)	94,340 (15.23)	94,194 (15.12)
31–35	114,274 (18.17)	115,156 (18.51)	117,753 (18.68)	115,047 (18.82)	117,911 (19.04)	119,583 (19.19)
36–40	109,745 (17.45)	110,546 (17.77)	113,424 (18.00)	114,374 (18.71)	118,752 (19.17)	121,393 (19.48)
41–45	140,234 (22.30)	131,724 (21.17)	125,199 (19.86)	117,550 (19.23)	115,326 (18.62)	114,872 (18.44)

Percentages may not total to 100 due to rounding

*n* number

### Screening for precancerous lesions

Between 2013 and 2018, the annual proportion of screened women ranged from 60.01% to 61.33 (Table 2). The highest annual proportions were found in women aged 27–30 years (69.36% in 2013 and 67.35% in 2018) and 31–35 years (68.64% in 2013 and 66.75% in 2018).

**Table 2:** Annual proportion of screened women aged 18–45 years from 2013 to 2018 in Germany

	Calendar year										
Age (in years)	2013		2014		2015		2016		2017		2018
Women in the database (total), *n* (%)											
18–45	628,755 (100.00)	622,120 (100.00)		630,284 (100.00)		611,380 (100.00)		619,416 (100.00)		623,040 (100.00)	
Women undergoing screening for precancerous lesions, *n* (% of women in the respective year, 95% CI)											
18–45	385,622 (61.33, 61.21–61.45)	381,436 (61.31, 61.19–61.43)		382,319 (60.66, 60.54–60.78)		369,176 (60.38, 60.26–60.51)		371,683 (60.01, 59.88–60.13)		374,081 (60.04, 59.92–60.16)	
Women undergoing screening for precancerous lesions stratified by age groups, *n* (% of women in the respective year and age group, 95% CI)											
18–19	210 (0.60, 0.52–0.69)	201 (0.57, 0.49–0.65)		185 (0.49, 0.42–0.57)		146 (0.40, 0.34–0.48)		141 (0.40, 0.33–0.47)		112 (0.31, 0.26–0.37)	
20–26	86,806 (60.86, 60.61–61.12)	86,035 (61.01, 60.75–61.26)		86,765 (60.23, 59.98–60.49)		81,808 (59.78, 59.52–60.04)		80,851 (58.82, 58.56–59.08)		81,087 (59.23, 58.97–59.49)	
27–30	60,287 (69.36, 69.05–69.67)	61,283 (69.50, 69.19–69.80)		63,552 (68.92, 68.62–69.22)		62,336 (68.16, 67.86–68.46)		64,058 (67.90, 67.60–68.20)		63,436 (67.35, 67.05–67.65)	
31–35	78,432 (68.64, 68.37–68.90)	78,705 (68.35, 68.08–68.62)		80,011 (67.95, 67.68–68.21)		77,376 (67.26, 66.98–67.53)		78,787 (66.82, 66.55–67.09)		79,817 (66.75, 66.48–67.01)	
36–40	72,031 (65.63, 65.35–65.92)	72,353 (65.45, 65.17–65.73)		73,746 (65.02, 64.74–65.30)		74,384 (65.04, 64.76–65.31)		76,477 (64.40, 64.13–64.67)		78,180 (64.40, 64.13–64.67)	
41–45	87,856 (62.65, 62.40–62.90)	82,859 (62.90, 62.64–63.16)		78,060 (62.35, 62.08–62.62)		73,126 (62.21, 61.93–62.49)		71,369 (61.88, 61.60–62.17)		71,449 (62.20, 61.92–62.48)	

*n* number; *CI* confidence interval

### CIN2+ diagnoses

The overall annual proportion of women with CIN2+ records (irrespective of screening participation) remained relatively stable over time and ranged from a minimum of 0.72% (2018) to a maximum of 0.84% (2014) in women 18–45 years old (Table 3). Mean age of women with prevalent CIN2+ diagnoses was 33.2 (SD: 6.9) years in 2013 and 34.3 (SD: 6.2) years in 2018.

**Table 3:** Annual proportions of prevalent CIN2+ records in women aged 18–45 years from 2013 to 2018 in Germany

Age (in years)	Calendar year					
	2013	2014	2015	2016	2017	2018
Women in the database (total), *n* (%)						
18–45	628,755 (100.00)	622,120 (100.00)	630,284 (100.00)	611,380 (100.00)	619,416 (100.00)	623,040 (100.00)
Women with CIN2+ records, *n* (% of women in the respective year, 95% CI)						
18–45	5,018 (0.80, 0.78–0.82)	5,256 (0.84, 0.82–0.87)	5,060 (0.80, 0.78–0.83)	4,649 (0.76, 0.74–0.78)	4,605 (0.74, 0.72–0.77)	4,489 (0.72, 0.70–0.74)
Women with CIN2+ records stratified by age groups, *n* (% of women in the respective year and age group, 95% CI)						
18–19	62 (0.18, 0.14–0.23)	53 (0.15, 0.11–0.20)	60 (0.16, 0.12–0.21)	47 (0.13, 0.10–0.17)	38 (0.11, 0.08–0.15)	45 (0.12, 0.09–0.17)
20–26	912 (0.64, 0.60–0.68)	876 (0.62, 0.58–0.66)	764 (0.53, 0.49–0.57)	612 (0.45, 0.41–0.48)	554 (0.40, 0.37–0.44)	476 (0.35, 0.32–0.38)
27–30	960 (1.10, 1.04–1.18)	1,029 (1.17, 1.10–1.24)	988 (1.07, 1.01–1.14)	873 (0.95, 0.89–1.02)	797 (0.84, 0.79–0.91)	751 (0.80, 0.74–0.86)
31–35	1,162 (1.02, 0.96–1.08)	1,292 (1.12, 1.06–1.18)	1,272 (1.08, 1.02–1.14)	1,174 (1.02, 0.96–1.08)	1,263 (1.07, 1.01–1.13)	1,280 (1.07, 1.01–1.13)
36–40	935 (0.85, 0.80–0.91)	1,036 (0.94, 0.88–1.00)	1,070 (0.94, 0.89–1.00)	1,075 (0.94, 0.88–1.00)	1,074 (0.90, 0.85–0.96)	1,072 (0.88, 0.83–0.94)
41–45	987 (0.70, 0.66–0.75)	970 (0.74, 0.69–0.78)	906 (0.72, 0.68–0.77)	868 (0.74, 0.69–0.79)	879 (0.76, 0.71–0.81)	865 (0.75, 0.70–0.80)

*n* number; *CI* confidence interval; *CIN* cervical intraepithelial neoplasia

**Fig. 2:**
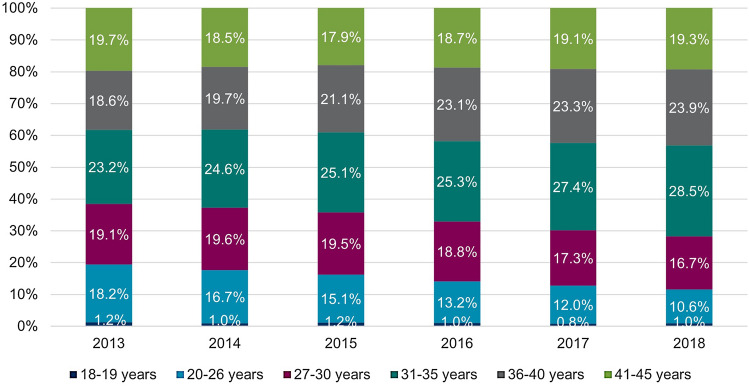
Distribution of women aged 18–45 years with prevalent CIN2+ records stratified by age groups. Abbreviations: *CIN*, cervical intraepithelial neoplasia

There were differences respect to the age groups with shifts over time (Table 3): The highest proportions in 2013 were found among 27–30-year-old (1.10%), and in 2018 among 31–35-year-old women (1.07%). For the age group 27–30 years, proportions decreased over time from 1.10% in 2013 to 0.80% in 2018 (Table 3). A similar observation was made for 20–26-year-old women (2013: 0.64%; 2018: 0.35%). For women aged 31 and older no such observation was made, instead, some proportion slightly increased Table 3. Over 60% of all 18–45-year-old women with prevalent CIN2+ diagnoses were between 31 and 45 years old. This share increased from 61.46% in 2013 to 71.66% in 2018 (Fig. 2).

**Fig. 3:**
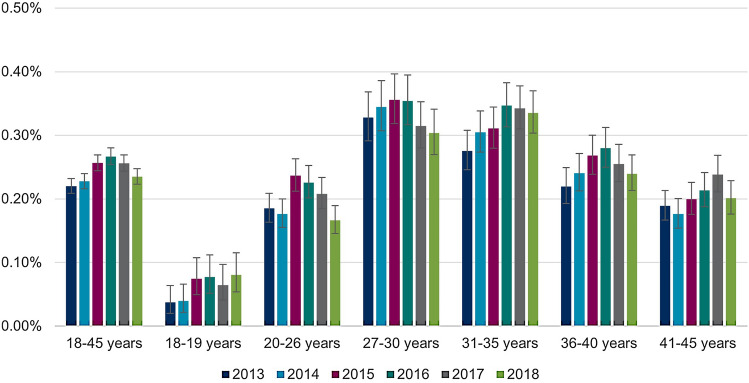
Annual proportions of prevalent CIN2 records by age group. Abbreviations: *CIN*, cervical intraepithelial neoplasia

**Fig. 4:**
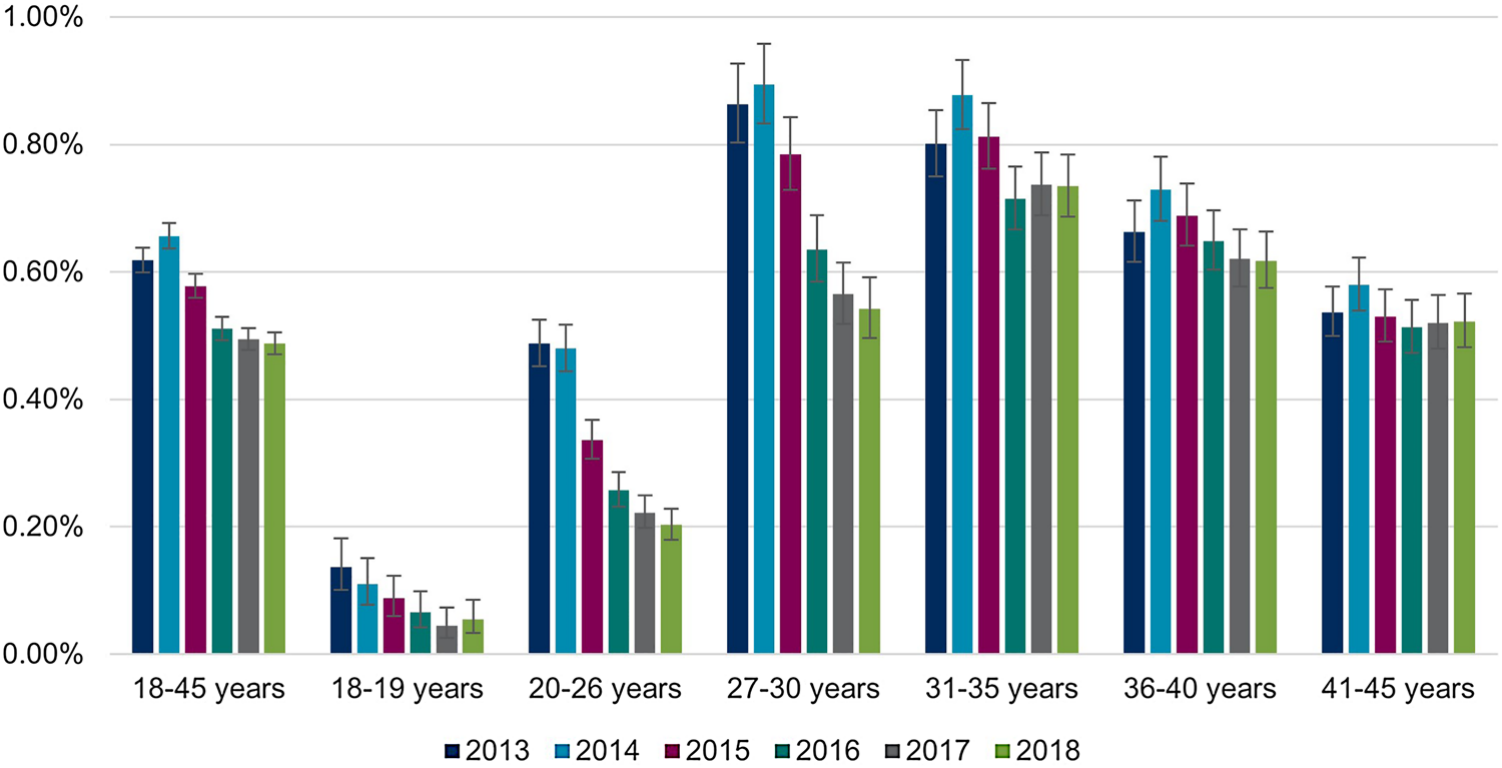
Annual proportions of prevalent CIN3 records by age group. Abbreviations: *CIN*, cervical intraepithelial neoplasia

Separate analyses of the annual proportions of women with prevalent CIN2 and CIN3 records showed higher interannual variability in proportions of women with CIN3 than in proportions of women with CIN2 diagnoses, primarily among women below the age of 31 (see Figs. 3 and 4). Mean age of women with CIN2 records was 33.0 (SD: 6.9) years in 2013 and 32.9 (SD: 6.6) years in 2018. Mean age of women with CIN3 diagnoses was 33.2 (SD: 6.9) years in 2013 and 34.6 (SD: 6.0) years in 2018.

**Fig. 5:**
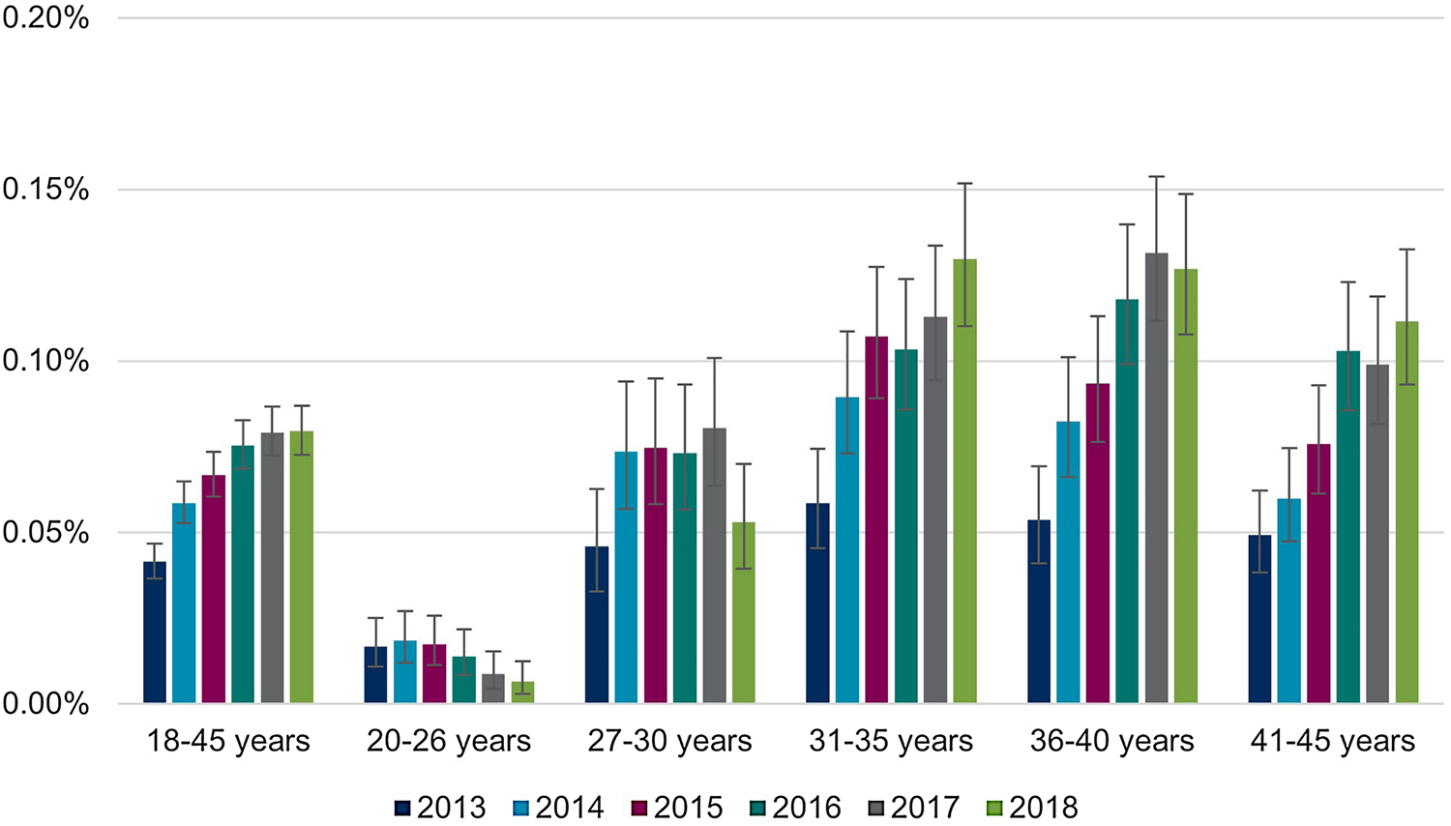
Annual proportions of prevalent cervical cancer records by age group. Please note that age group 18–19 was left out of the figure due to data protection regulations, as patient counts were either 0 or 1–4 in all years

Annual proportions of women with prevalent cervical cancer records are shown in Fig. 5. Highest proportion was observed in 2018 in women 31 and older. Mean age of women with cervical cancer was 35.2 (SD: 6.4) years in 2013 and 36.5 (SD: 5.1) years in 2018.

The annual proportions of women aged 18–45 years with incident CIN2+ records were 0.35%, 0.34%, and 0.32% in 2016, 2017, and 2018, respectively. In general, the tendencies of variations over years and age groups for incident CIN2+ records are similar to those observed for prevalent CIN2+ records. More details on annual proportions of women with incident CIN2+ records, can be found in the Supplement, Section 5.

### Cervical conization

**Fig. 6:**
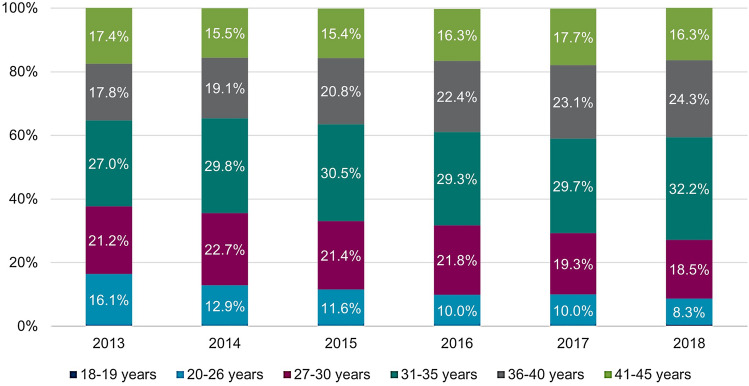
Annual distribution of age groups among women aged 18–45 years undergoing cervical conization

**Fig. 7:**
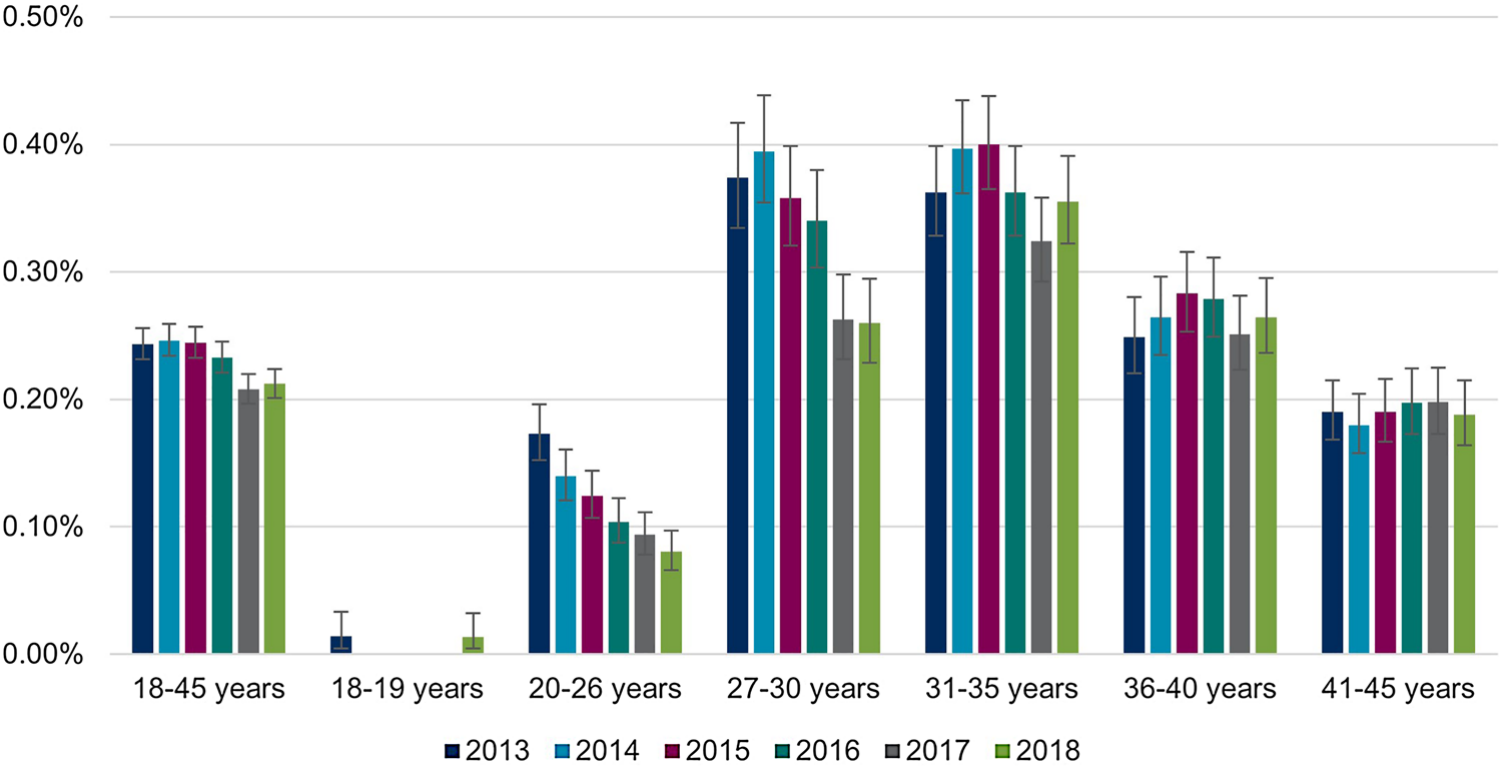
Annual proportions of women undergoing conization by age groups. Please note that for the years 2014–2017, age group 18–19 years has been left out of the figure due to data protection regulations, as patient counts were 1–4

The annual proportions of women undergoing conization are shown in Table 4. There were differences by age group and over time similar to those reported for the annual CIN2+ prevalence. Over 60% of all observed women with a claim for cervical conization were 31–45 years old (Fig. 6). The annual proportion of women undergoing conization declines from 0.24% in 2013 to 0.21% in 2018 (Table 4 & Fig. 6). As seen in proportions of women with CIN2+ records, declines over time were seen in younger age groups 20–26 (0.17% in 2013 vs. 0.08% in 2018) and 27–30 (0.37% in 2013 vs. 0.26% in 2018). Proportions in age groups above the age of 30 years appeared stable over time (Fig. 7).

**Table 4:** Annual proportion of women aged 18–45 years undergoing conization from 2013 to 2018 in Germany

Age (in years)	Calendar year					
	2013	2014	2015	2016	2017	2018
Women in the database (total), *n* (%)						
18–45	628,755 (100.00)	622,120 (100.00)	630,284 (100.00)	611,380 (100.00)	619,416 (100.00)	623,040 (100.00)
Women undergoing cervical conization, total n in the database *(% of women in the respective year, 95% CI)*						
18–45	1,531 (0.24, 0.23–0.26)	1,532 (0.25, 0.23–0.26)	1,542 (0.24, 0.23–0.26)	1,425 (0.23, 0.22–0.25)	1,288 (0.21, 0.20–0.22)	1,322 (0.21, 0.20–0.22)
Women undergoing cervical conization, total n in the database stratified by age groups (% of women in the respective age group and year, 95% CI)						
18–19	5 (0.01, 0.00–0.03)	< 5 (−)	< 5 (−)	< 5 (−)	< 5 (−)	5 (0.01, 0.00–0.03)
20–26	247 (0.17, 0.15–0.20)	197 (0.14, 0.12–0.16)	179 (0.12, 0.11–0.14)	142 (0.10, 0.09–0.12)	129 (0.09, 0.08–0.11)	110 (0.08, 0.07–0.10)
27–30	325 (0.37, 0.33–0.42)	348 (0.39, 0.35–0.44)	330 (0.36, 0.32–0.40)	311 (0.34, 0.30–0.38)	248 (0.26, 0.23–0.30)	245 (0.26, 0.23–0.29)
31–35	414 (0.36, 0.33–0.40)	457 (0.40, 0.36–0.43)	471 (0.40, 0.36–0.44)	417 (0.36, 0.33–0.40)	382 (0.32, 0.29–0.36)	425 (0.36, 0.32–0.39)
36–40	273 (0.25, 0.22–0.28)	292 (0.26, 0.23–0.30)	321 (0.28, 0.25–0.32)	319 (0.28, 0.25–0.31)	298 (0.25, 0.22–0.28)	321 (0.26, 0.24–0.29)
41–45	267 (0.19, 0.17–0.21)	237 (0.18, 0.16–0.20)	238 (0.19, 0.17–0.22)	232 (0.20, 0.17–0.22)	228 (0.20, 0.17–0.23)	216 (0.19, 0.16–0.21)

*n* number; *CI* confidence interval

### Subsequent records of CIN and conization

Overall, the subpopulation used for analysis of subsequent records of CIN and repeated conization comprised N = 2,749 women. Please see Supplement, Section 5 for detailed description of the patient selection process as well as age statistics.

Of these women, 1,156 (42.05%) received a subsequent CIN (1–3+) record in the 24-months follow-up period (CIN records in the six weeks after index conization were not considered). For more information on subsequent CIN (1–3+) records please see Supplement, Section 5.

For the assessment of re-conizations, only conizations performed 90 days after the index conization were considered. After applying the wash-out period of 90 days, 80 (2.91%) women underwent at least one subsequent conization after index conization in the 24-months follow-up period. On average, the first subsequent conization after the wash-out period of 90 days was performed 8.4 months (252.3 days, SD: 146.1) after the initial conization. The age group with the shortest mean duration until a subsequent conization (6.9 months, 206.7 days, SD: 79.0) was the age group 20–26 years. The age group with the longest mean duration until a subsequent conization was the age group 31–35 years with 10.1 months (303.0 days, SD: 192.9).

### Sensitivity analyses

The cross-sectional analyses of women with incident and prevalent of CIN2+ records and conizations were repeated restricting the study population to screened women instead of all women in the database.

The annual proportion of women with prevalent CIN2+ diagnoses in women aged 18–45 year with screening records in the same calendar year was 1.23% in 2013 and 1.12% in 2018 (Supplement, Section 6, Supplementary Table 4). The highest annual prevalence in 2013 was observed in age groups 27–30 years (1.53%) and 31–35 years (1.41%). In 2018, the highest prevalence was observed in the age group 31–35 years (1.50%) and 36–40 years (1.27%).

The annual proportions of women incident CIN2+ diagnoses in screened women from 2016 to 2018 can be found in Supplement, Section 6, Supplementary Table 5. Overall, the proportions decreased during the observation period (0.55% in 2016 and 0.50% in 2018).

The proportion of women undergoing conization among women aged 18–45 years screened for precancerous lesions in the same calendar year was 0.37% in 2013 and 0.34% in 2018 (Supplement, Section 6, Supplementary Table 6). Stratified by age, the highest proportions of women undergoing conization were found in the age group 27–30 years in 2013 and 2014 (0.52% and 0.56%, respectively) and in the age group 31–35 years in 2015–2018 (0.56%, 0.49%, 0.46% and 0.51%, respectively).

## Discussion

In this claims data analysis, we found a considerable CIN2+ prevalence and incidence as well as substantial burden of conization procedures in women aged 18–45 years in Germany.

Overall, annual screening proportions, CIN2+ prevalence, incidence, and conization proportions remained relatively stable over time in Germany showing only a slight decrease in 18–45-year-old women between 2013 and 2018. Annual proportions of screened women ranged from 61.33 to 60.01%, annual proportions or women with prevalent CIN2+ diagnoses ranged from 0.84 to 0.72% (irrespective of screening participation), annual proportions of women with incident CIN2+ diagnoses ranged from 0.35 to 0.32%, and annual proportions of women undergoing conization ranged from 0.25 to 0.21%. In almost 3% of women undergoing cervical conization a record for subsequent (re-)conization was observed during the timeframe of 3–24 months after an initial conization.

We found differences between age groups for the CIN2+ prevalence, incidence and conization proportions. Firstly, the highest burden occurred in women aged 27 years and older: The annual proportion of women with prevalent CIN2+ records was > 1% irrespective of screening participation in 31–35-year-old women in every year between 2013 and 2018 and in 27–30-year-old women in 2013, 2014 and 2015. Considering the results from the sensitivity analysis including only screened women (where the probability of undetected CIN should be considerably lower), annual proportion of women with CIN2+ records was as high as 1.5% (27–30-year-old women in 2014). Separate analyses of proportions by CIN grade revealed that the burden was mainly driven by CIN3 diagnoses. Second, in younger age groups below age 31, the burden decreased over time. The annual CIN2+ prevalence was 1.10% in 2013 and 0.80% in 2018 for the group of 27–30-year-old women. These changes were also apparent when only CIN3 diagnoses were considered. With these changes in younger age groups, the relative share of 31–45-year-old women in all women with a prevalent CIN2+ diagnoses increased from 61.46% in 2013 to 71.66% in 2018.

To our knowledge, the present study is the most comprehensive report on the burden of CIN2+ and conizations in Germany. Only very few other reports exist. In our previous study assessing the burden of HPV-associated anogenital diseases in young women using the same claims database, we have already reported a three-year CIN3 prevalence of 0.3% in 2013–2015 in women born in 1990 [21]. While the previous study only looked at 23–25-year-old women, the present study incorporates a broader age range from 18 to 45 years. Additionally, the focus in our present study was extended to cover not only CIN, but also conization outcomes and screening proportions. Previous results for frequency of annual conizations in Germany varied between 50,000 and 140,000 (based on 2006 to 2009 extrapolated estimates) [23, 26, 27]. Projecting the annual proportion of women undergoing a conization observed in our study in the InGef Research Database in 2013 (0.24%) and 2018 (0.21%) to the whole German population of 18–45 year-old women (approximately 13.7 and 13.6 million women in 2013 and 2018, respectively [35]), this would indicate 33,313 conizations in 2013 and 28,828 conizations in 2018 for 18–45-year-old women, which is lower than the range of previous reports. The lower numbers in our study are presumably mainly due to the age restriction, whereas the previously reported numbers did not make such restrictions. However, the reported annual rate of 217 conizations per 100,000 women, based on German claims data from 2009 is in line with our findings [23].

Our finding that around 3% of women needed a repeated conization during a two-year timeframe, implies that there was a high proportion of subsequent lesions, which is in line with previously reported recurrence proportions after treatment for CIN. An Italian retrospective analysis of recurrence of high-grade cervical lesions following LEEP for CIN2+ reported 5% within two years and 6% at 5 years [36]. Results from a nationwide Danish registry study also suggested that for women undergoing a conization due to CIN3, an increased risk for subsequent events remained [28]. Several mechanisms have been described to explain why women after treatment remain at increased risk of subsequent lesions and progression to cancer, including inadequate excision with positive surgical margins or persistent HPV infection [37], but also re-activation and predisposition to new HPV infections (reinfection, e.g., from an infected partner) have been suggested [38], which is methodologically difficult to study. However, the observed 42.1% of women with a subsequent CIN (1–3 +) record 43 days to 24 months after conization was not in a plausible range from a clinical perspective. Therefore, we assume that these records do not indicate recurrent CIN, but that CIN records are kept in medical records after a conization, probably for reasons of surveillance.

The observed burden of CIN2+ and (re-)conizations indicate a need for continued and better prevention of HPV infections in Germany. Although the most effective measure against HPV-associated diseases, such as CIN2+ and cervical cancer, is prophylactic vaccination [39], vaccination coverage rates in Germany have remained at relatively low levels compared to other countries (e.g., 77% HPV vaccine coverage in Australia and New Zealand [40]), despite the reported increases from 27.2% in 2011 to 43.0% in 2018 for a complete vaccination series in 15-year-old girls (51.1% for 18-year-old women in 2018) [41]. Attempts to further increase HPV vaccination rates and completion rates of vaccination series are needed. To achieve this, it is important to raise awareness in the population, e.g., through public information campaigns and active outreach to patients by physicians [42].

Our findings show that the highest proportions of women with incident CIN2+ were found in the age groups 27–30 years (0.50% in 2016) and 31–35 years (0.52% in 2017 and 0.48% in 2018) (see Supplement, Section 4 for detailed results). Given an estimated average duration from infection to CIN2+ development of 1–3 years, it can be assumed that incident infections might have been potentially prevented not only by vaccinating adolescents, but also by broader catch-up HPV vaccination coverage in adults [6, 7, 43]. Even though it is acknowledged by STIKO that women aged 18 and older may benefit from HPV vaccination [39], HPV vaccination is currently only reimbursed regularly up to age 17 in Germany. Selected health insurances cover costs for HPV vaccination up to age 26, but no data exist to what extent patients and physicians seize this opportunity.

Findings from other studies additionally suggest that HPV vaccination of women with CIN before or after conization may offer protection against subsequent CIN2+ occurrence, irrespective of age at vaccination [44, 45]. Thus, it could be argued that HPV vaccination should also be offered to women with CIN diagnoses, as they are at elevated risk for future HPV diseases [28, 29].

The reasons for the observed decline in CIN2+ prevalence and conization proportions over time in younger women below age 27 are unclear and warrant further investigation. Changes in screening, sexual behavior and treatment decisions need to be considered in addition to a potential impact of HPV vaccination. Screening guidelines have not changed in Germany during the study period and the annual proportion of screening in our study appeared relatively constant over time. Among screening-eligible age groups (20+), lowest annual proportions of screening participation were observed in 20–26-year-old women (approximately 60%). Highest annual participation proportions were observed in 27–30-year-old women (approximately 68%). German clinical practice has shifted in more recent years toward an adoption of watch and wait strategies instead of immediate conization for CIN2 [23]. This may have contributed to a decreased conization burden particularly in younger women, given reported high regression rates in these age groups [23, 46, 47]. However, we observed changes in both conization proportions and CIN2+ burden, arguing against a strong impact of treatment decisions. Introduction and uptake of HPV vaccination in adolescent girls after 2007 may have also contributed to the prevention of CIN2+ and therefore the decline in CIN2+ and conizations in younger women. Women < 30 years in our study population were eligible for reimbursed HPV vaccination as adolescents. HPV vaccine coverage rates are not known for the study population but are, as outlined previously, generally rather low in Germany (51% for complete schedule in 18-year-old girls in 2018) [41].

## Limitations

The present study has some limitations, most inherent with the use of health insurance claims data. Claims data are primarily collected for reimbursement purposes. Only patients who see a physician and cause reimbursement for the health insurance can be identified in the database. For the identification of CIN2+ we used ICD-10-GM codes. To ensure the accuracy of diagnoses only “verified” diagnoses in the outpatient and primary and secondary diagnoses in the inpatient sector were used. With this approach however, we may have excluded women whose “suspected” diagnosis had yet to be confirmed. This could have led to an underestimation of CIN prevalence and incidence. For the records of conization, we deliberately focused on specific OPS codes for cervical conization, including LEEP and LEETZ (5–671.0* or 5–671.1*) as these are the recommended procedures for CIN treatment in Germany [23]. However, the German OPS catalog additionally lists codes for “other excision and destruction of cervical tissue” (OPS code 5–672*). It is possible that in some cases this code was documented instead of the code for conization, leading to an underestimation of performed procedures related to CIN. Overall, our results must be seen as capturing the administrative burden which may differ from the actual clinical burden, as our analyses were based on secondary data, more precisely on codes primarily recorded for reimbursement as opposed to purely scientific purposes.

Moreover, the burden of CIN2+ may have been underestimated, as it is a disease that can only be detected by screening. To approximate the true clinical burden more closely, we assessed screening proportions and CIN diagnoses in the subpopulation of screened women as a sensitivity analysis. In this analysis, CIN2+ prevalences were higher (1.12% in 2018) compared to prevalences in the overall population (0.72% in 2018). We nevertheless focused primarily on the results in the overall population irrespective of screening participation because the assessment of screening participation posed some additional limitations. First, there is no code for cervical cancer screening as such. Consequently, every record of a Pap or HPV DNA test was considered as indicator for screening, even though these tests may also be done as part of the diagnostic work-up or follow-up. Furthermore, our analysis did not consider the chronological sequence of screening and CIN diagnosis or conization events, respectively, within the respective calendar year of analysis. Therefore, it remains unclear if screening followed the initial diagnosis of CIN or the initial conization as a method of follow-up or if screening preceded the respective diagnosis and indication for conization.

For the incidence assessment, we used a slightly simplified concept for cumulative incidence calculation by not dividing by the number of individuals at risk but by the total number of observable women in the database. We chose to use this simplified approach as the true number of women at risk was not identifiable in the database (e.g., incomplete information on former hysterectomies). We expect this approach to be conservative as it might slightly underestimate true incidence, however, we do not expect underestimation to be large, as the condition is relatively rare and therefore there should not be a large difference in denominators between this simple and more sophisticated approaches. Also, we do not expect this simplification to bias description of differences between years as all observed years should be equally affected.

The follow-up time of 2 years to investigate subsequent CIN and subsequent (re-)conizations after initial conization is relatively short considering the average time from infection to CIN diagnosis or conization of 1–3 years reported in the literature [5–7]. Additionally, we introduced a wash-out period of 90 days (i.e., 3 months) after the index conization for the assessment of re-conizations. All conization codes recorded during this period were not considered as (re-)conizations. This conservative approach was deemed reasonable because clinical and histologic post-conization follow-up is unlikely to result in earlier re-conizations due to persistent or recurrent CIN. Likely reasons for earlier re-conizations would be post-operative hemorrhage, which we did not evaluate with the chosen method. Hence overall, we might have underestimated the actual proportion of women undergoing re-conizations.

Our analysis of subsequent CIN records has particular limitations that we want to highlight. The observed 42.1% of women with a subsequent CIN (1–3 +) record is clinically not plausible, particularly with 33% being CIN3 records. Recurrence rates of high-grade CIN have previously been reported with approximately 5% [36]. It was not possible from the claims data to differentiate unequivocally between new CIN diagnoses after an index conization or perpetuated initial diagnostic coding related to documentation of post-operative surveillance. We used a wash-out period of 6 weeks (42 days) after index conizations for the analysis of subsequent CIN records to mitigate this limitation, but still this might have been too short.

Our study included only adult women aged 18–45 years. This age range was chosen because the main disease burden was expected in this age group. However, the burden of CIN2+ and conization was still substantial in the oldest age group (41–45 years) and it can be expected that there is an existing disease burden also in women beyond age 45.

## Conclusion

In sum, a substantial burden of CIN2+ and conizations remains in Germany with the highest burden in women older than 27 years, indicating the need for intensified prevention efforts. Increasing HPV vaccination rates in adolescents, combined with intensified catch-up vaccination of adult women could be an important measure to reduce the burden of CIN diagnoses. This would also reduce the number of conizations as well as potential negative psychological and medical effects associated with diagnosis and treatment of CIN.

## Supplementary Information

Below is the link to the electronic supplementary material.

Supplementary file1 (DOCX 131 KB)

## Data Availability

The data used in this study cannot be made available in the manuscript, the supplemental files, or in a public repository due to German data protection laws (Bundesdatenschutzgesetz). To facilitate the replication of results, anonymized data used for this study are stored on a secure drive at the Institute for Applied Health Research Berlin (InGef) GmbH. Access to the data used in this study can only be provided to external parties under the conditions of the cooperation contract of this research project and can be assessed upon request, after written approval at InGef GmbH (Tel. + 49 (30) 21 23 36–471; info@ingef.de), if required.
