# Supplementary material for: Burden of CIN2+ diagnoses and conizations in women aged 18–45 years—a retrospective secondary data analysis of German statutory health insurance claims data
Source: Arch Gynecol Obstet. 2022 Apr 14;306(6):2077–92. doi: 10.1007/s00404-022-06548-7 (PMC9633516; doi:10.1007/s00404-022-06548-7)
Supplement: Supplementary file 1 — Supplementary file1 (DOCX 131 KB) [file 404_2022_6548_MOESM1_ESM.docx]

**Supplementary information**

**Burden of CIN2+ Diagnoses and Conizations in Women aged 18-45 Years - A Retrospective Secondary Data Analysis of German Statutory Health Insurance Claims Data**

Archives of Gynecology and Obstetrics

Miriam Reuschenbach^1*^, Anna-Janina Stephan^2*^, Kunal Saxena^3^, Vimalanand S. Prabhu^3^, Christian Jacob^4^, Kim Maren Schneider^4^, Wolfgang Greiner^5^, Regine Wölle^2^, Monika Hampl^6^

^1^ Global Medical and Scientific Affairs, MSD Sharp & Dohme GmbH, Munich, Germany

^2^ Department of Market Access, MSD Sharp & Dohme GmbH, Munich, Germany

^3^ Center for Observational and Real-World Evidence (CORE), Merck & Co., Inc., Kenilworth, NJ, USA

^4^ EU Real World Evidence, Xcenda GmbH, Hanover, Germany

^5^ Department of Health Economics and Health Care Management, Bielefeld School of Public Health, Bielefeld University, Bielefeld, Germany

^6^ Department of Gynecology, University of Duesseldorf, Duesseldorf, Germany

*These authors contributed equally to this work

**Corresponding Author:**

Miriam Reuschenbach

MSD Sharp & Dohme GmbH, Levelingstr. 4a, 81673 Munich, Germany

Email: [miriam.reuschenbach@msd.de](mailto:miriam.reuschenbach@msd.de)

Content

[1 Database description 3](#_Toc93308042)

[2 Description of matching process 4](#_Toc93308043)

[3 Identification of screening, CIN, and cervical conization 6](#_Toc93308044)

[4 Annual proportions of women with incident CIN2+ records 8](#_Toc93308045)

[5 Subgroup analysis – subsequent CIN records 10](#_Toc93308046)

[6 Sensitivity analysis 11](#_Toc93308047)

# Database description

The Institute for Applied Health Research Berlin (InGef) consists of about 8 million covered lives and includes the healthcare resource utilization and costs of services in an anonymized case-by-case individual format. For scientific research projects, an adjusted analysis sample of the InGef database has been created which comprises anonymized claims data of about 4 million individuals from about 60 SHI companies, thereby covering about 55% of all SHI companies in Germany^[[1]](#footnote-1)^, 5.5% of the German SHI population and 4.8% of the total German population in 2018. The database represents the German population in terms of age and gender according to the Federal Office of Statistics^[[2]](#footnote-2)^. It has proven to have good external validity to the German population in terms of morbidity, mortality, and drug use^[[3]](#footnote-3)^. Available data include patient demographics, inpatient and outpatient diagnoses according to the International Statistical Classification of Diseases, German Modification (ICD-10-GM), healthcare resource utilization as well as costs for inpatient care (according to the Operation and Procedure Classification System (Operationen- und Prozedurenschlüssel, OPS), the German modification of the International Classification of Procedures in Medicine (ICPM), which includes surgery and diagnostics codes) and for outpatient care (coded through the uniform assessment standard (Einheitlicher Bewertungsmaßstab, EBM), the ambulatory doctors fee schedule in Germany), pharmacological therapy (standardized documentation numbers and Anatomical Therapeutic Chemical (ATC) classification codes), as well as remedies and aids (Germany-specific standardized documentation numbers), and sick leave in an anonymized case-by-case format. The InGef Research Database covers a maximum span of six consecutive years of data and is updated on an annual basis leading to an annual addition of the most recent and an annual removal of the least recent year of data from the database. At the time of this analysis, 2013-2018 data were available.

# Description of matching process

For the assessment of subsequent CIN records as well as subsequent (re-)conizations, we used an existing subsample (termed “study cohort 1”) of CIN patients undergoing surgical treatment (conization) from the same InGef study population that was created in the matching process for a comparative analysis of women with CIN undergoing conization, women with CIN but without conization and women with neither CIN nor conization for another part of this study which has been published elsewhere. For the sake of reproducibility, we describe the process that produced this sample and was used in this paper for analysis of subsequent CIN records and re-conizations (study cohort 1), even though the results of the actual primary objective from this matching analysis are not part of this paper.

The overall study population was drawn from the InGef Research Database using data from 2013-2018 for all women aged 18-45 years between 2013-2016.

*Study population eligible for study cohort 1: CIN patients undergoing surgical treatment (conization)*

From the overall study population (18–45-year-old women in 2013-2016), all women were identified if they had at least one diagnosis for CIN (1/2/3) in the timeframe from January 1^st^, 2013, until December 31^st^, 2016. Thereof, all women with a medical record for conization (index event) within six months after a CIN diagnosis in the timeframe from July 1^st^, 2013, until December 31^st^, 2016, were identified. The identified women needed to be continuously observable for at least 24 months beginning with the index event and six months prior conization (baseline period) to assess baseline demographics and baseline clinical characteristics including a record of most severe CIN diagnosis. In addition, no record for conization, re-conization or cervical cancer (ICD-10-GM code C53.-) in the six months baseline period was allowed.

*Study population eligible for study cohort 2: CIN patients with conservative management*

From the overall study population (18–45-year-old women in 2013-2016), all women in the InGef Research Database were identified if they had at least one diagnosis for CIN (1/2/3) in the timeframe from January 1^st^, 2013, until December 31^st^, 2016. The identified women in study cohort 2 were not allowed to have any record for cervical conization or re-conization or cervical cancer (ICD-10-GM code C53.-) in the complete study period (January 1^st^, 2013, until December 31^st^, 2018). Therefore, study cohort 2 needed to be continuously observable for the complete study period (January 1^st^, 2013, until December 31^st^, 2018).

*Study population eligible for control group: Women with neither CIN diagnoses nor conization*

From the overall study population (18–45-year-old women in 2013-2016), all women in the InGef Research Database were identified if they neither had a diagnosis for CIN (1/2/3), cervical cancer (ICD-10-GM code C53.-) nor a medical record for cervical conization in the complete study period (January 1^st^, 2013, until December 31^st^, 2018). Therefore, the identified women needed to be continuously observable for the entire study period from January 1^st^, 2013, until December 31^st^, 2018.

A 1:1:1 direct, exact matching without replacement between women with CIN and conization (study cohort 1) and women with CIN who did not undergo a conization (study cohort 2) versus women without both CIN diagnoses and conization (control group) was performed. Study cohort 2 was matched on age and most severe CIN grade in the same year-specific quarter to study cohort 1. The control group was matched on age in the respective calendar year to both study cohorts. To define a common follow-up period for all three study groups, the date of the index event of study cohort 1 (conization after CIN diagnosis) was assigned to both study cohort 2 and the control group as a virtual index event to mark the start of the 24 months follow-up period.

Overall, this matching process produced a sample of women with CIN records undergoing conization and observable for at least 24 months post conization (study cohort 1) that was suitable for analysis of subsequent records of CIN and (re-)conization and comprised N=2,749 women. The mean age of eligible women in study cohort was 33.6 (SD: 6.1) years after the matching. More details on the age distribution of this subsample can be found in Supplementary Table 1.

Supplementary Table 1 Age distribution in women eligible for study cohort 1 during the six-month baseline period before index event – after matching

|  | Study cohort 1 | |
| --- | --- | --- |
|  | n | % |
| Total 18-45-year-old women | 2,749 | 100.00 |
| Mean age (standard deviation) | 33.60 (6.11) | |
| Age groups |  |  |
| 18-19 years | <5 | - |
| 20-26 years | 345 | 12.55 |
| 27-30 years | 598 | 21.75 |
| 31-35 years | 764 | 27.79 |
| 36-40 years | 577 | 20.99 |
| 41-45 years | 461 | 16.77 |

# Identification of screening, CIN, and cervical conization

Supplementary Table 2 Codes used for the identification of screening, CIN, and cervical conization

| Code | Variable | English description | German description |
| --- | --- | --- | --- |
| EBM code | | | |
| 01730 | Pap test | Examination for the early detection of cancer | Untersuchung zur Früherkennung von Krebserkrankungen bei der Frau gemäß Abschnitt B. II. §§ 6 und 8 der Krebsfrüherkennungs-Richtlinie |
| 01733 | Pap test | Cytological examination | Zytologische Untersuchung gemäß Abschnitt B. II. §§ 7 und 8 der Krebsfrüherkennungs-Richtlinie  Obligater Leistungsinhalt:  - Zytologische Untersuchung eines oder mehrerer Abstriche, auch Bürstenabstriche, von Ekto- und/oder Endozervix  Fakultativer Leistungsinhalt:  - Abstrichentnahme von Ekto- und/oder Endozervix, einschl. Kosten |
| 32819 | HPV test | DNA detection exclusively of high-risk HPV types from a body material | DNA-Nachweis ausschließlich von High-Risk-HPV-Typen aus einem Körpermaterial (Direktnachweis) mittels Hybridisierung ggf. einschließlich Aufbereitung und/oder Amplifikation (z. B. Nukleinsäureisolierung, -denaturierung, -transfer) bei Zustand nach operativem (operativen) Eingriff(en) an der Cervix uteri wegen CIN I bis CIN III |
| 32820 | HPV test | DNA and/or mRNA detection exclusively of high-risk HPV types from a cervical/vaginal material | DNA- und/oder mRNA-Nachweis ausschließlich von High-Risk-HPV-Typen aus einem Zervix- /Vaginalmaterial mittels sequenzspezifischen Nachweises ggf. einschl. Aufbereitung und/oder Amplifikation nur bei einem Zervixzytologiebefund ab Gruppe III nach Münchner Nomenklatur III |
| ICD-10-GM code | | | |
| N87.0 | CIN I | Low-grade dysplasia of the cervix uteri  Low-grade squamous intraepithelial lesion (LSIL)  Cervical intraepithelial neoplasia [CIN], grade I | Niedriggradige Dysplasie der Cervix uteri  Niedriggradige squamöse intraepitheliale Läsion [LSIL]  Zervikale intraepitheliale Neoplasie **[CIN] I**. Grades |
| N87.1 | CIN II | Medium grade dysplasia of the cervix uteri  Cervical intraepithelial neoplasia [CIN], grade II | Mittelgradige Dysplasie der Cervix uteri  Zervikale intraepitheliale Neoplasie **[CIN] II**. Grades |
| N87.2 | CIN III | High-grade dysplasia of the cervix uteri, not elsewhere classified | Hochgradige Dysplasie der Cervix uteri, anderenorts nicht klassifiziert |
| D06.- |  | Carcinoma in situ of cervix uteri  Cervical intraepithelial neoplasia [CIN], grade III, with or without mention of severe dysplasia | Carcinoma in situ der Cervix uteri Zervikale intraepitheliale Neoplasie **[CIN] III**. Grades, mit oder ohne Angabe einer hochgradigen Dysplasie |
| C53.- | Cervical Cancer | Malignant neoplasm of cervix uteri | Bösartige Neubildung der Cervix uteri |
| OPS code | | | |
| 5-671.0 | Conization | Conization | Konisation |
| 5-671.00 | Laser excision | Laser excision | Laserexcision |
| 5-671.01 | Loop excision | Loop excision | Schlingenexcision |
| 5-671.02 | Knife excision | Knife excision | Messerkonisation |
| 5-671.03 | Electrical needle/knife excision | Electrical needle/knife excision | Excision mit elektrischer Nadel/Messer |
| 5-671.0x | Other | Other | Sonstige |
| 5-671.1 | Re-conization | Re-conization | Rekonisation |
| 5-671.10 | Laser excision | Laser excision | Laserexcision |
| 5-671.11 | Loop excision | Loop excision | Schlingenexcision |
| 5-671.12 | Knife excision | Knife excision | Messerkonisation |
| 5-671.13 | Electrical needle/knife excision | Electrical needle/knife excision | Excision mit elektrischer Nadel/Messer |
| 5-671.1x | Other | Other | Sonstige |
| Abbreviations: *CIN* cervical intraepithelial neoplasia, *EBM* Official German Remuneration Scheme for Outpatient Care [Einheitlicher Bewertungsmaßstab], *einschl.* einschließlich, *ggf.* gegebenenfalls, *HPV* human papillomavirus, ICD-10-GM International Statistical Classification of Diseases, 10th Revision, German Modification, *LSIL* low grade squamous intraepithelial lesion [niedriggradige squamöse intraepitheliale Läsion], *mRNA* messenger ribonucleic acid, *OPS* German classification of operation and procedures [Operations- und Prozedurenschlüssel] *z.B.* zum Beispiel. | | | |

# Annual proportions of women with incident CIN2+ records

The proportion of women aged 18-45 years with incident CIN2+ diagnoses was 0.35%, 0.34%, and 0.32% in 2016, 2017, and 2018, respectively (see Supplementary Table 3). The highest proportions were found among age group 27-30 years in 2016 (0.50%) and 31-35 years in 2017 (0.52%). Mean age of women with incident CIN2+ diagnoses was 33.4 (SD: 6.7) years in 2016, 33.8 (SD: 6.5) years in 2017, and 33.5 (SD: 6.4) years in 2018.

Supplementary Table 3 Annual proportions of women with incident CIN2+ records in women aged 18-45 years from 2016-2018 in Germany

|  | Calendar year | | |
| --- | --- | --- | --- |
| Age  (in years) | 2016 | 2017 | 2018 |
| Women in the database (total), n (%) | | | |
| 18-45 | 611,380  (100.00) | 619,416  (100.00) | 623,040  (100.00) |
| Women with 4-year observability timeframe, including three years before plus the respective year of interest in the database, n (%) | | | |
| 18-45 | 476,930  (100.00) | 474,145  (100.00) | 475,380  (100.00) |
| Women with incident CIN2+ diagnoses, total n in the database *(% of women of the same 4-year observability timeframe in the respective year and age group, 95% CI)* | | | |
| 18-45 | 1,685  *(0.35, 0.34-0.37)* | 1,607  *(0.34, 0.32-0.36)* | 1,518  *(0.32, 0.30-0.34)* |
| Women with incident CIN2+ diagnoses stratified by age groups, total n in the database *(% of women of the same 4-year observability timeframe in the respective year and age group, 95% CI)* | | | |
| 18-19 | 26  *(0.09, 0.06-0.13)* | 13  *(0.04, 0.02-0.08)* | 25  *(0.09, 0.06-0.13)* |
| 20-26 | 258  *(0.26, 0.23-0.29)* | 226  *(0.23, 0.20-0.26)* | 195  *(0.20, 0.17-0.23)* |
| 27-30 | 319  *(0.50, 0.44-0.55)* | 255  *(0.39, 0.35-0.44)* | 276  *(0.42, 0.38-0.48)* |
| 31-35 | 394  *(0.45, 0.40-0.49)* | 460  *(0.52, 0.47-0.57)* | 429  *(0.48, 0.44-0.53)* |
| 36-40 | 391  *(0.42, 0.38-0.46)* | 341  *(0.36, 0.32-0.40)* | 346  *(0.36, 0.32-0.40)* |
| 41-45 | 297  *(0.29, 0.26-0.33)* | 312  *(0.32, 0.29-0.36)* | 247  *(0.26, 0.22-0.29)* |

Abbreviations: *n*, number; *CI*, confidence interval; *CIN*, cervical intraepithelial neoplasia

Results of the separate analyses of women with incident CIN2 and CIN3 diagnoses are shown in see Fig. 1 and Fig. 2, respectively. The proportion of women aged 18-45 years with incident CIN2 diagnoses was 0.11%, 0.10%, and 0.09% in 2016, 2017, and 2018, respectively. In comparison, the proportion of CIN3 diagnoses was more than twice as high (0.23%, 0.23%, and 0.22% in 2016, 2017, and 2018, respectively). Mean age of women with incident CIN2 diagnoses was 32.5 (SD: 7.0) years in 2016 and 32.3 (SD: 6.8) years in 2018. Mean age of women with incident CIN3 diagnoses was 33.5 (SD: 6.6) years in 2016 and 33.8 (SD: 6.2) years in 2018.

Fig. 1 Annual proportion of women with incident CIN2 records by age group

Abbreviations: *CIN*, cervical intraepithelial neoplasia

Fig. 2 Annual proportion of women with incident CIN3 records by age group

Abbreviations: *CIN*, cervical intraepithelial neoplasia

# Subgroup analysis – subsequent CIN records

Of the 2,749 women in the subsample used for analysis of subsequent records for CIN and (re‑)conization, 1,156 (42.05%) received at least one CIN (1-3+) record in the 24-months follow-up period (CIN records in the six weeks after index conization were not considered). Women could have multiple subsequent diagnoses during the follow-up, i.e., if a women presented with CIN2 and CIN3 diagnoses in the follow-up, she was considered for both proportions of women with a least one CIN2+ diagnosis and women with at least one CIN3 diagnosis. The most frequently identified subsequent record in these women was CIN3 (33.43%) (Fig. 3). Furthermore, 9.24% of women were diagnosed with CIN2, 5.24% with CIN1 and 0.44% with cervical cancer.

Fig. 3 Frequency of patients with at least one subsequent CIN record six weeks after index conization within 24 months follow-up

Abbreviations: *CIN*, cervical intraepithelial neoplasia

# Sensitivity analysis

Screened women (using HPV test/Pap test) with prevalent CIN2+ diagnoses were identified by using specific codes (see Supplementary Table 2). Screened women with at least one outpatient “verified” diagnosis or inpatient primary or secondary diagnosis for CIN2+ in the respective calendar year were considered as screened women with CIN2+. The annual proportion of women with prevalent CIN2+ diagnoses was calculated by dividing the number of screened women with a prevalent CIN2+ diagnosis in that calendar year by the total number of screened women in the respective calendar year.

Screened women with incident CIN2+ diagnosis were identified by using ICD-10-GM codes for CIN2+ diagnosis (see Supplementary Table 2). Screened women with at least one outpatient “verified” diagnoses or inpatient primary or secondary diagnoses for CIN2+ in the respective calendar but no diagnosis of any kind in a period of three calendar years before the analysis year were considered as screened women with incident CIN2+. The annual proportion of women with incident CIN2+ diagnoses was calculated by dividing the number of screened women with an incident CIN2+ diagnosis in that calendar year by the total number of screened women in that calendar year (only for 2016, 2017, and 2018), who were also continuously observable three years prior to the respective analysis year.

Screened women (using HPV test/Pap test) with at least one recorded claim of the listed OPS codes for cervical conization (see Supplementary Table 2) in the respective calendar year were defined as screened women who underwent a cervical conization. The annual proportion of women undergoing conization in screened women was calculated by dividing the number of screened women with a record of conization in that calendar year by the total number of screened women in the respective calendar year.

Supplementary Table 4 Annual proportions of screened women with prevalent CIN2+ stratified by age groups

|  | Calendar year | | | | | |
| --- | --- | --- | --- | --- | --- | --- |
| Age  (in years) | 2013 | 2014 | 2015 | 2016 | 2017 | 2018 |
| Women in the database (total), n | | | | | | |
| 18-45 | 628,755 | 622,120 | 630,284 | 611,380 | 619,416 | 623,040 |
| Women undergoing screening for precancerous lesions, n *(% of women in the respective year)* | | | | | | |
| 18-45 | 385,622 *(61.33)* | 381,436 *(61.31)* | 382,319 *(60.66)* | 369,176 *(60.38)* | 371,683 *(60.01)* | 374,081 *(60.04)* |
| Women undergoing screening for precancerous lesions and with prevalent CIN2+, total n in the database *(% of women in the respective age group and year, 95% CI)* | | | | | | |
| 18-45 | 4,743  *(1.23, 1.20-1.27)* | 4,967  *(1.30, 1.27-1.34)* | 4,737  *(1.24, 1.20-1.27)* | 4,324  *(1.17, 1.14-1.21)* | 4,305  *(1.16, 1.12-1.19)* | 4,175  *(1.12, 1.08-1.15)* |
| Women undergoing screening for precancerous lesions and with prevalent CIN2+ stratified by age groups, total n in the database *(% of women in the respective age group and year, 95% CI)* | | | | | | |
| 18-19 | 31  *(14.76, 10.26-20.29)* | 24  *(11.94, 7.80-17.24)* | 22  *(11.89, 7.60-17.45)* | 20  *(13.70, 8.57-20.36)* | 18  *(12.77, 7.74-19.42)* | 15  *(13.39, 7.69-21.13)* |
| 20-26 | 865  *(1.00, 0.93-1.06)* | 827  (0.96, 0.90-1.03) | 712  *(0.82, 0.76-0.88)* | 564  *(0.69, 0.63-0.75)* | 519  *(0.64, 0.59-0.70)* | 447  *(0.55, 0.50-0.60)* |
| 27-30 | 920  *(1.53, 1.43-1.63)* | 993  *(1.62, 1.52-1.72)* | 940  *(1.48, 1.39-1.58)* | 835  *(1.34, 1.25-1.43)* | 759  *(1.18, 1.10-1.27)* | 715  *(1.13, 1.05-1.21)* |
| 31-35 | 1,102  *(1.41, 1.32-1.49)* | 1,222  *(1.55, 1.47-1.64)* | 1,198  *(1.50, 1.41-1.58)* | 1,082  *(1.40, 1.32-1.48)* | 1,170  *(1.49, 1.40-1.57)* | 1,195  *(1.50, 1.41-1.58)* |
| 36-40 | 883  *(1.23, 1.15-1.31)* | 975  *(1.35, 1.26-1.43)* | 1,006  *(1.36, 1.28-1.45)* | 1,007  *(1.35, 1.27-1.44)* | 1,*009*  *(1.32, 1.24-1.40)* | 994  *(1.27, 1.19-1.35)* |
| 41-45 | 942  *(1.07, 1.01-1.14)* | 926  *(1.12, 1.05-1.19)* | 859  *(1.10, 1.03-1.18)* | 816  *(1.12, 1.04-1.19)* | 830  *(1.16, 1.09-1.24)* | 809  *(1.13, 1.06-1.21)* |

Abbreviations: *CI* confidence interval, *CIN* cervical intraepithelial neoplasia.

Supplementary Table 5 Annual proportions of screened women with incident CIN2+ stratified by age groups

|  | |  | | Calendar year |  |
| --- | --- | --- | --- | --- | --- |
| Age  (in years) | | 2016 | | 2017 | 2018 |
| Women in the database (total), n | | | | | |
| 18-45 | 611,380 | | | 619,416 | 623,040 |
| Women undergoing screening for precancerous lesions and continuously observable three years prior to the respective analysis year, n *(% of women in the respective year)* | | | | | |
| 18-45 | | | 289,423 *(47.34)* | 285,248 *(46.05)* | 285,796 *(45.87)* |
| Women undergoing screening for precancerous lesions and with incident CIN2+, total n in the database *(% of women in the respective age group and year, 95% CI)* | | | | | |
| 18-45 | | | 1,595  *(0.55, 0.52-0.58)* | 1,529  *(0.54, 0.51-0.56)* | 1,441  *(0.50, 0.48-0.53)* |
| Women undergoing screening for precancerous lesions and with incident CIN2+, n *(% of women in the respective age group and year, 95% CI)* | | | | | |
| 18-19 | 16  *(13.56, 7.95-21.08)* | | | 7  *(6.73, 2.75-13.38)* | 11  *(12.50, 6.41-21.27)* |
| 20-26 | 242  *(0.41, 0.36-0.46)* | | | 216  *(0.38, 0.33-0.43)* | 185  *(0.32, 0.28-0.37)* |
| 27-30 | 309  *(0.70, 0.62-0.78)* | | | 247  *(0.55, 0.49-0.63)* | 268  *(0.61, 0.54-0.68)* |
| 31-35 | 365  *(0.61, 0.55-0.67)* | | | 432  *(0.72, 0.66-0.79)* | 413  *(0.69, 0.62-0.76)* |
| 36-40 | 379  *(0.61, 0.55-0.68)* | | | 331  *(0.53, 0.48-0.59)* | 330  *(0.52, 0.47-0.58)* |
| 41-45 | 284  *(0.45, 0.40-0.50)* | | | 296  *(0.49, 0.43-0.54)* | 234  *(0.39, 0.34-0.44)* |
|  |  | | |  |  |

Supplementary Table 6 Annual proportions of screened women undergoing conization stratified by age groups

|  | Calendar year | | | | | |
| --- | --- | --- | --- | --- | --- | --- |
| Age  (in years) | 2013 | 2014 | 2015 | 2016 | 2017 | 2018 |
| Women in the database (total), n | | | | | | |
| 18-45 | 628,755 | 622,120 | 630,284 | 611,380 | 619,416 | 623,040 |
| Women undergoing screening for precancerous lesions, n *(% of women in the respective year)* | | | | | | |
| 18-45 | 385,622 *(61.33)* | 381,436 *(61.31)* | 382,319 *(60.66)* | 369,176 *(60.38)* | 371,683 *(60.01)* | 374,081 *(60.04)* |
| Women undergoing both screening for precancerous lesions and cervical conization, total n in the database *(% of women in the respective age group and year, 95% CI)* | | | | | | |
| 18-45 | 1,446  *(0.37, 0.36-0.39)* | 1,*460*  *(0.38, 0.36-0.40)* | 1,470  *(0.38, 0.37-0.40)* | 1,338  *(0.36, 0.34-0.38)* | 1,217  *(0.33, 0.31-0.35)* | 1,264  *(0.34, 0.32-0.36)* |
| Women undergoing both screening for precancerous lesions and cervical conization stratified by age groups, total n in the database *(% of women in the respective age group and year, 95% CI)* | | | | | | |
| 18-19 | <5  *(-)* | 0  *(0.0, 0.0-1.82)* | 0  *(0.0, 0.0-1.97)* | <5  *(-)* | <5  (-) | <5  (-) |
| 20-26 | 235  *(0.27, 0.24-0.31)* | 189  *(0.22, 0.19-0.25)* | 172  *(0.20, 0.17-0.23)* | 130  *(0.16, 0.13-0.19)* | 121  *(0.15, 0.12-0.18)* | 108  *(0.13, 0.11-0.16)* |
| 27-30 | 311  *(0.52, 0.46-0.58)* | 342  *(0.56, 0.50-0.62)* | 313  *(0.49, 0.44-0.55)* | 300  *(0.48, 0.43-0.54)* | 238  *(0.37, 0.33-0.42)* | 235  *(0.37, 0.32-0.42)* |
| 31-35 | 395  *(0.50, 0.46-0.56)* | 435  *(0.55, 0.50-0.61)* | 451  *(0.56, 0.51-0.62)* | 382  *(0.49, 0.45-0.55)* | 359  *(0.46, 0.41-0.51)* | 408  *(0.51, 0.46-0.56)* |
| 36-40 | 252  *(0.35, 0.31-0.40)* | 270  *(0.37, 0.33-0.42)* | 307  *(0.42, 0.37-0.47)* | 308  *(0.41, 0.37-0.46)* | 281  *(0.37, 0.33-0.41)* | 307  *(0.39, 0.35-0.44)* |
| 41-45 | 252  *(0.29, 0.25-0.32)* | 224  *(0.27, 0.24-0.31)* | 227  *(0.29, 0.25-0.33)* | 217  *(0.30, 0.26-0.34)* | 217  *(0.30, 0.26-0.35)* | 204  *(0.29, 0.25-0.33)* |

1. GKV Spitzenverband. *Die gesetzlichen Krankenkassen. Anzahl der Krankenkassen im Zeitablauf - Konzentrationsprozess durch Fusionen (Angaben am Stichtag 1. 1.)*. 2019 5. Aug 2018]; Available from: <https://www.gkv-spitzenverband.de/krankenversicherung/kv_grundprinzipien/alle_gesetzlichen_krankenkassen/alle_gesetzlichen_krankenkassen.jsp>. [↑](#footnote-ref-1)
2. Statistisches Bundesamt DESTATIS. *Ergebnisse der Bevölkerungsfortschreibung auf Grundlage des Zensus 2011*. 2019; Available from: <https://www.destatis.de/DE/ZahlenFakten/GesellschaftStaat/Bevoelkerung/Bevoelkerungsstand/Tabellen/Zensus_Geschlecht_Staatsangehoerigkeit.html>. [↑](#footnote-ref-2)
3. Andersohn, F. and J. Walker, *Characteristics and external validity of the German Health Risk Institute (HRI) Database.* Pharmacoepidemiol Drug Saf, 2016. **25**(1): p. 106-9. [↑](#footnote-ref-3)
